# HIV-1 Env DNA Vaccine plus Protein Boost Delivered by EP Expands B- and T-Cell Responses and Neutralizing Phenotype In Vivo

**DOI:** 10.1371/journal.pone.0084234

**Published:** 2013-12-31

**Authors:** Kar Muthumani, Megan C. Wise, Kate E. Broderick, Natalie Hutnick, Jonathan Goodman, Seleeke Flingai, Jian Yan, Chaoran B. Bian, Janess Mendoza, Colleen Tingey, Christine Wilson, Krzysztof Wojtak, Niranjan Y. Sardesai, David B. Weiner

**Affiliations:** 1 Department of Pathology and Laboratory Medicine, University of Pennsylvania, Philadelphia, Pennsylvania, United States of America; 2 Inovio Pharmaceuticals Inc, Blue Bell, Pennsylvania, United States of America; Federal University of São Paulo, Brazil

## Abstract

An effective HIV vaccine will most likely require the induction of strong T-cell responses, broadly neutralizing antibodies (bNAbs), and the elicitation of antibody-dependent cellular cytotoxicity (ADCC). Previously, we demonstrated the induction of strong HIV/SIV cellular immune responses in macaques and humans using synthetic consensus DNA immunogens delivered via adaptive electroporation (EP). However, the ability of this improved DNA approach to prime for relevant antibody responses has not been previously studied. Here, we investigate the immunogenicity of consensus DNA constructs encoding gp140 sequences from HIV-1 subtypes A, B, C and D in a DNA prime-protein boost vaccine regimen. Mice and guinea pigs were primed with single- and multi-clade DNA via EP and boosted with recombinant gp120 protein. Sera were analyzed for gp120 binding and induction of neutralizing antibody activity. Immunization with recombinant Env protein alone induced low-titer binding antibodies with limited neutralization breath. In contrast, the synthetic DNA prime-protein boost protocol induced significantly higher antibody binding titers. Furthermore, sera from DNA prime-protein boost groups were able to neutralize a broader range of viruses in a panel of tier 1 clade B viruses as well as multiple tier 1 clade A and clade C viruses. Further investigation of synthetic DNA prime plus adaptive EP plus protein boost appears warranted.

## Introduction

There is an urgent need for improved vaccination approaches against HIV that induce improved humoral and cellular immune responses [1]–[4]. It is generally agreed upon that strong T-cell responses and breath in neutralizing antibodies will likely play a role in the development of a protective vaccine [1], [4]–[6]. Though DNA platforms in the past have been poor inducers of seroconversion [7], [8], recent improvements in construct design, improved delivery, and improved formulations have enhanced the immune potency of this approach [7], [9]–[11]. We have recently reported the induction of strong HIV/SIV-specific cellular immune responses in mice, macaques and humans using consensus DNA immunogens delivered via electroporation (EP) [7], [9], [12]–[15]. While these studies have confirmed the induction of a potent and broad cell-mediated response, the ability of this improved DNA-EP platform to induce or prime for neutralizing antibodies (NAbs) is unknown. Due to a heightened interest in trying to improve immune responses to HIV included by DNA prime-protein boost vaccination strategies, here we studied this combination focused on increasing binding titers and neutralization capacity *in vivo* [2], [3], [16].

In this study, we examined the immunogenicity of a synthetic consensus DNA vaccine encoding gp140 constructs derived from individual HIV-1 subtypes A, B, C and D in a DNA prime-protein boost regimen. These consensus DNA constructs were optimized using the following plasmid-enhancement techniques: codon optimization, RNA optimization, leader sequence addition, plasmid production at high concentrations and the DNA was delivered by adaptive EP as previously described [13], [14], [17]–[20]. The DNA prime was followed by a protein boost with recombinant HIV gp120. Immune responses were measured by ELISA, B-cell ELISpot, T-cell ELISpot, and in a TZM-Bl neutralization assay. The combination approach increased T cell and antibody functionality over these observed with either independent modality.

## Materials and Methods

### Ethics Statement

Mice were housed and treated in a temperature-controlled, light-cycled facility in accordance with the guidelines of the National Institutes of Health (Bethesda, MD, USA) and the University of Pennsylvania (Philadelphia, PA, USA) Institutional Animal Care and Use Committee (IACUC #801577).

Female Dunkin-Hartley guinea pigs weighing between 350 and 450 g and free of intercurrent infection were obtained from Charles River (Wilmington, MA) and were housed at Bio-Quant, Inc., (San Diego, CA) through collaboration with Inovio Pharmaceuticals Inc., PA. The protocol was approved by the Committee on the Ethics of Animal Experiments at the Bio-Quant, Inc. In collaboration with the animal resource dept. of Inovio Pharmaceuticals Inc., (Number: 08–021). At all locations, animals were handled based on the recommendations in the Guide for the Care and Use of Laboratory Animals of the National Institutes of Health. In accordance with the Weatherall report, animal welfare was ensured and steps were taken to ameliorate or minimize.

### Cells and Reagents

HeLa, 293T and Jurkat (ATCC, Manassas, VA) and HeLa-CD4-TZM-bl (NIH-AIDS Reagent Program, MD) cells were maintained in Dulbecco's modified Eagle's medium (DMEM; Gibco-Invitrogen) supplemented with 10% Fetal Bovine Serum (FBS) and antibiotics and passaged upon confluence [13], [19], [21]. Recombinant HIV-1 envelope gp120 proteins were obtained from Protein Sciences Corporation (Meriden, CT) and peroxidise-conjugated streptavidin from Jackson Laboratory. HIV-1 envelope polyclonal antibodies and other viral reagents were obtained through the AIDS Research and Reference Reagent Program, Division of AIDS, NIAID, NIH (Germantown, MD).

### HIV-1 Envelope construction and expression

The HIV-1 envelope consensus sequences were previously described [22]–[25]. Briefly, consensus sequences of HIV-1 envelope from subtype clades A, B, C and D were constructed with modifications as discussed [19], [26]–[28]. An IgE leader sequence was added to all envelope antigen sequences to improve expression, and the cytoplasmic tail was truncated to prevent envelope recycling. The resulting optimized HIV-Env DNA immunogens were codon and RNA-optimized, and balanced for GCAT content and synthesized [19], [24], [29], [30], and cloned into the modified pVax1 expression vector [19], [24]. Large-scale amplification of DNA constructs were carried out by Aldevron (Fargo, ND), and purified plasmid DNA was formulated in water for immunization as described [9].

To test the expression, cells were transfected for expression analysis of synthetic envelopes using the non-liposomal FuGENE transfection reagent (Roche Applied Science, Indianapolis, IN) as suggested by the manufacturer. Briefly, cells were seeded at 70% confluence (50,000 cells per well in 6-well plates) a day before and transfected with 5 μg of the HIV-1 envelope plasmids. Cells were harvested 48 to 72 hrs post-transfection in 1× RIPA buffer (50 mM Tris/HCl (pH 7.4), 150 mM NaCl, 1% Triton X-100, 1% sodium deoxycholate, and 0.1% SDS, supplemented with a complete protease inhibitor cocktail from Roche Applied Science, Indianapolis, IN) and mixed with SDS sample buffer (0.08M Tris (pH 6.8); 2.0% SDS, 10% glycerol, 0.1 M dithiothreitol, 0.2% bromophenol blue) before boiling for 5 minutes.

For envelope expression study by immunoblot, specific sera or Abs were diluted 1∶100 in PBS and reacted with individual strips for 1 h. Subsequently, strips were washed four times with Tris-buffered saline- 0.2% Tween, reacted with a peroxidase-coupled antiserum against mouse IgG (Sigma, St Louis, MO), and incubated with diaminobenzidine substrate (Sigma, St. Louis, MO) [31]. For immunofluorescent analysis of optimized envelope expression, RD cells were transfected with vaccine constructs and stained with monoclonal antibodies against HIV-1 envelope (4E10; NIH-AIDS Reagent Program, MD) and against MHC-I prior to confocal analysis. All images were captured by Zeiss 710 LSM Meta microscope system observed in PBS [18].

### Mice and guinea pig immunizations

Study 1 was performed in the BALB/c mice, using four groups of animals (4 animals per group/repeated 3 times) which received DNA or protein immunogens alone or in prime-boost combinations. Study 2, for guinea pig study (5 animals/group; repeated 2 times), HIV-1 Env plasmids were immunized. Animals in prime-boost and recombinant groups received HIV-1 clade B protein (50 μg) formulated with TiterMax adjuvant (Sigma-Aldrich, St Louis, MO) at weeks 8 and 11. A sham immunization group received an empty pVax1 vector and sham protein (PBS) formulated with TiterMax.

At various time points, small amounts of peripheral blood was harvested from the mice and guinea pigs for analysis of the humoral immune response using ELISA assay. For analysis of the cellular immune response, the mice were humanely sacrificed at week 7 and their spleens were used as a source of lymphocytes for the ELISpot and flow cytometry immune analysis. Immunizations were delivered into the quadriceps muscles by *in vivo* Cellectra^®^-adaptive EP as described previously [7], [19], [28], [32]. All procedures were performed in accordance with the guidelines of the National Institutes of Health (Bethesda, MD, USA) and the University of Pennsylvania (Philadelphia, PA, USA) Institutional Animal Care and Use Committee.

### ELISpot assay

We determined antigen-specific T-cell responses via IFN-**γ** ELISpot. Briefly, ELISpot 96-well plates (EMD Millipore Corporation, Billerica, MA) were coated with anti-mouse IFN-**γ** capture Abs and incubated for 24 h at 4°C (R&D Systems, Minneapolis, MN). The following day, plates were washed and blocked for 2 h with 1% BSA. Splenocytes (10^5^) from the immunized mice were added to each well and stimulated overnight at 37°C in 5% CO_2_ in the presence of RPMI 1640 (negative control), Concanavalin A (positive control), or specific peptide pools (10 μg/ml). Peptide pools consist of 15-mer peptides overlapping by 11 amino acids. After 24 h of stimulation, the cells were washed and incubated for 24 h at 4°C with biotinylated anti-mouse IFN-**γ** Abs (R&D Systems, Minneapolis, MN). The plates were washed, and streptavidin–alkaline phosphatase (R&D Systems, Minneapolis, MN) was added to each well and incubated for 2 h at room temperature. The plates were then washed and 5-bromo-4-chloro-3′-indolylphosphate p-toluidine salt and nitro blue tetrazolium chloride (chromogen color reagent; R&D Systems, Minneapolis, MN) were added to each well. The plates were then rinsed with distilled water and dried at room temperature.

When testing for antibody-secreting B cells (ASCs), splenocytes were not stimulated prior to detection by ELISpot assay, but instead were tested directly after isolation from the spleen. MultiScreen-IP plates (Millipore, Billerica, MA) were coated with affinity-purified goat anti-mouse IgG (KPL, Gaithersburg, MD) in PBS. Plates were washed six times with PBS and blocked with RPMI with 10% FCS for 2 h at room temperature. Splenocytes (10^5^) were added to each well of the ELISpot plate in at least 100 μl of medium and incubated overnight at 37°C. Plates were washed six times in PBS with 0.25% Tween 20 (Sigma-Aldrich, St. Louis, MO) (PBS-T) and incubated with 100 μl of 1∶5,000 biotin-IgG (Jackson ImmunoResearch Laboratories, Inc., West Grove, PA) for 1 h at room temperature. Plates were then washed and incubated with 100 μl of 1∶60 streptavidin-AP (R&D Research Systems, Minneapolis, MN) for 1 h at room temperature. The plates were washed with PBS-T, PBS, and distilled water and developed with 100 μl of BCIP/NBT (R&D Research Systems, Minneapolis, MN) for 20 min at room temperature; the reaction was stopped with distilled water.

ELISpot spots were counted by an automated ELISpot reader (CTL Limited, Shaker Heights, OH). Raw values were determined and multiplied by the appropriate factor so that the data could be represented as SFC or ASCs per million splenocytes [33].

### Intracellular cytokine staining (ICS)

The phenotype of the responding T cells were analyzed by ICS fluorescence-activated cell sorting (FACS) analysis as described [14], [32]. Splenocytes (5×10^6^) were stimulated with a 15-mer HIV-1 MN Env peptide pool (2 μg/ml of each peptide; NIH AIDS Research & Reference Reagent Program, MD). BD GolgiStop™ & BD GolgiPlug™ (BD Biosciences, San Jose, CA) was added during the final 4 h of incubation. Cells were then stained with anti-mouse CD16/32 (Fc block) antibody, followed by surface staining. Cells were surface stained with CD3-FITC, CD4-Alexa 700, CD8-PerCP (BD Biosciences, San Jose, CA). Cells were then fixed, permeabilized with cytofix cytoperm (BD Biosciences, San Jose, CA) and stained with anti- IFN-γ-PE and anti-IL2-FITC. One million cells per sample were acquired on a BD LSRII flow cytometer (BD Biosciences, San Jose, CA) and CD4+ and CD8+ events (gated previously on CD3+ cells) were gated versus IFN-γ and IL-2. Sample analysis was performed using FlowJo software (Tree Star, Ashland, OR).

### Antibody binding assay: ELISA

For antibody detection, mouse serum samples were collected 7 days after the last immunization. Standard ELISAs were performed using HIV-1 recombinant gp120 as the antigen source, which was prepared as previously described [21], [34]. Antibody binding assays were carried out with either individual animal or pooled sera from the mice or Guinea pig in each group immunized with DNA or DNA+gp120 from different clades of HIV. Briefly, high-binding polystyrene Corning® 96 well plates (Sigma, St Louis, MO) were coated overnight at 4°C with recombinant envelope protein (MN clade B) (2 μg/ml) (Protein Sciences Corporation, Meriden, CT), which was diluted in 50 mM carbonate buffer (pH 9.6) and stored overnight at 4°C.

The next day, plates were washed with PBS-T (PBS, 0.05% Tween 20) and blocked for 1 h with 3% BSA in PBS-T. Bound IgG was detected using goat anti-mouse IgG-HRP (Sigma, St Louis, MO) at a dilution of 1∶5,000. Bound enzyme were detected by the addition of the chromogen substrate solution TMB (R&D Systems, Minneapolis, MN). The enzymatic reaction was stopped with 1N H_2_SO_4_ and plates were read at a 450-nm wavelength on a Biotek EL312e Bio-Kinetics reader (BioTek, Winooski, VT). All samples were assayed in triplicate. To determine the titers of antibodies after the last immunization, the sera from mice within a group were pooled, serially diluted, and analyzed by ELISA as described above. All samples were assayed in triplicate. End-point titers were calculated as the highest dilution, resulting in a reading of two OD above the value of a negative control serum [32], [35], [36].

### HIV-1 single-round pseudovirus production

The HIV-1 proviral infectious DNA construct pNL4–3/ΔEnv, and primary clade- specific envelope isolates were obtained through the AIDS Research and Reference Reagent (RRR)-Program, National Institute of Health (NIH). Pseudoviruses were produced using the pNL4–3/ΔEnv DNA plasmid encoding the HIV backbone and a plasmid encoding multiple primary as well as laboratory viruses, including clade A (92RW020) clade B (MN/H9; Bal.26; 6535.3, SF162 LS; NL4–3 and HXB2); clade C (MW965.26); and clade A/G (DJ263) envelope variants as previously described [37]. HIV-1 pseudoviral particles were generated by transfection with pNL4–3/ΔEnv alone or co-transfection with a panel of envelope plasmids by FuGENE 6 transfection in 293T cells. Two to three days after transfection, virion-containing culture supernatants were harvested, pre-cleared by centrifugation at 1,200RPM for 7 min and filtered through a 0.45-μm-pore-size membrane. Cleared culture supernatants were then treated with DNase I (Roche Applied Science, Indianapolis, IN) at a final concentration of 20 μg/ml at 37°C for 1 h and aliquots in 300 μl fractions were saved at −80°C until needed. The p24 concentrations of the virus stocks were quantified by HIV-1 p24 antigen ELISA as described [38], [39]. Titration of pseudotyped virus was determined using the 50% tissue culture infectious dose (TC-ID50) assay in TZM-BI cells described [21].

### HIV-1 neutralization assay

Neutralization titers were measured as a function of the reduction in luciferase reporter gene expression after a single round of viral infection in TZM-Bl cells as previously described [40]. TZM-Bl cells were obtained through the NIH AIDS Research and Reference Reagent Program. These cells are engineered to express CD4 and CCR5 and contain integrated reporter genes for firefly luciferase and *E. coli* β-galactosidase under control of an HIV-1 LTR.

Guinea pig sera were heat-inactivated at 56°C for 1 h prior to the assay. 25 μl of sera from the four groups were diluted in 125 μl of PBS. The diluted sera were further diluted threefold in a 96-well plate. Fifty microliters of a cell-free virus (200 TCID_50_) were added to each well. After 1 h of incubation, a 10,000 TZM-Bl cell suspension was added to each well. The plates were incubated for 48 h, after which 20 μl of lysis buffer (Cell Culture Lysis Reagent, Promega. Madison, WI) was added to each well at room temperature for 10 min and followed by 100 μl of Bright-Glo substrate and buffer (Luciferase Assay system, Promega, Madison, WI). The plate was read immediately with Glomax Luminometer (Promega, Madison, WI). The percentages of RLU reduction were calculated as (1−(average RLU of duplicates with sample sera−control wells)/(average RLU from mock control sera−control wells)) ×100%. Neutralizing antibody titers were expressed as the reciprocal of the serum dilution required to reduce the RLU by 50% [37].

### Statistical analysis

Group analyses were completed by matched, two-tailed, unpaired *t*-test and all values are mean ± SEM. Statistical analysis was performed using the Graph Pad Prism v.5.0 program (GraphPad Software, Inc., San Diego, CA). P-values of p<0.05 are considered to be statistically significant.

## Results

### Designs of HIV-1 envelope consensus DNA vaccines

**Figure 1:**
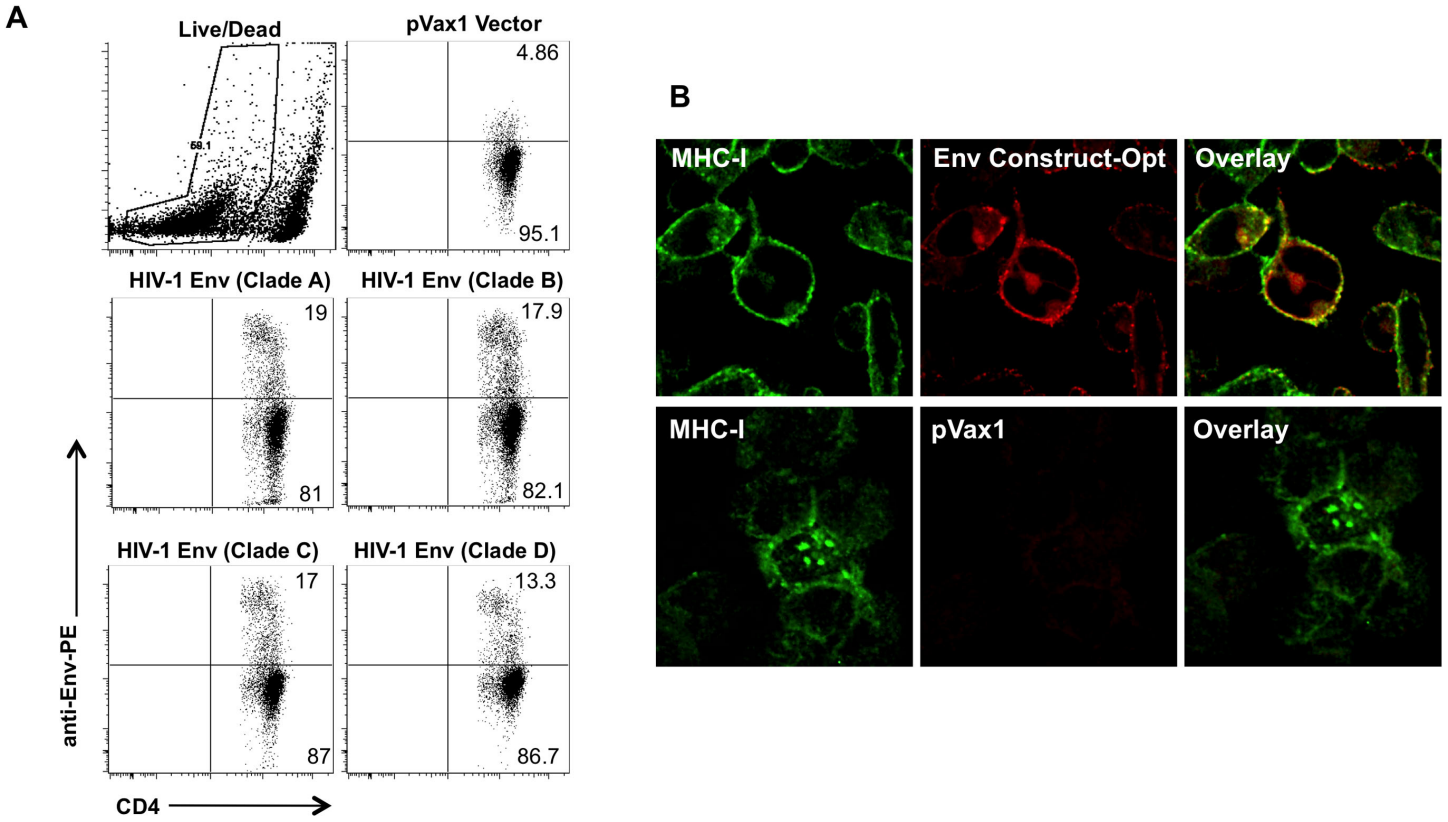
Optimized HIV-1 Env protein expression. (A) Jurkat T-cells were transfected with an HIV-1 Env-expressing plasmid and expression was determined by FACS. Cells were stained with anti-HIV core or control antibodies followed by PE-conjugated goat anti-mouse and human CD4-FITC treatment. (B) Immunofluorescent analysis of optimized envelope expression. Human RD cells were transfected with vaccine constructs and stained with monoclonal antibodies against MHC-I and HIV-1 Env prior to confocal analysis. MHC-I was used as a marker for cell surface expression as it is ubiquitously expressed on the surface of nucleated mammalian cells.

HIV-1 envelope was constructed as previously described [22], [30], [41]. Several modifications were made in constructing the plasmid including addition of a highly efficient IgE leader peptide sequence to facilitate expression. The resulting Env plasmid expressed at high levels in tissue culture as shown by FACS analysis in Jurkat cells (Figure 1A). To verify the expression of the consensus HIV-1 Env plasmids, an indirect immuno fluorescence assay (IFA) with confocal microscopy of transfected RD cells was performed, using a anti-HIV-1 Env 4E10 mAbs antibody, we observed colocalization of the specific cell membrane expression of Env with MHC-1 expression (Figure 1B). No expression was detected in control vector (pVax1) transfected cells.

### Improved CD8 T-cell responses by optimized HIV-1 envelope plasmid

**Figure 2:**
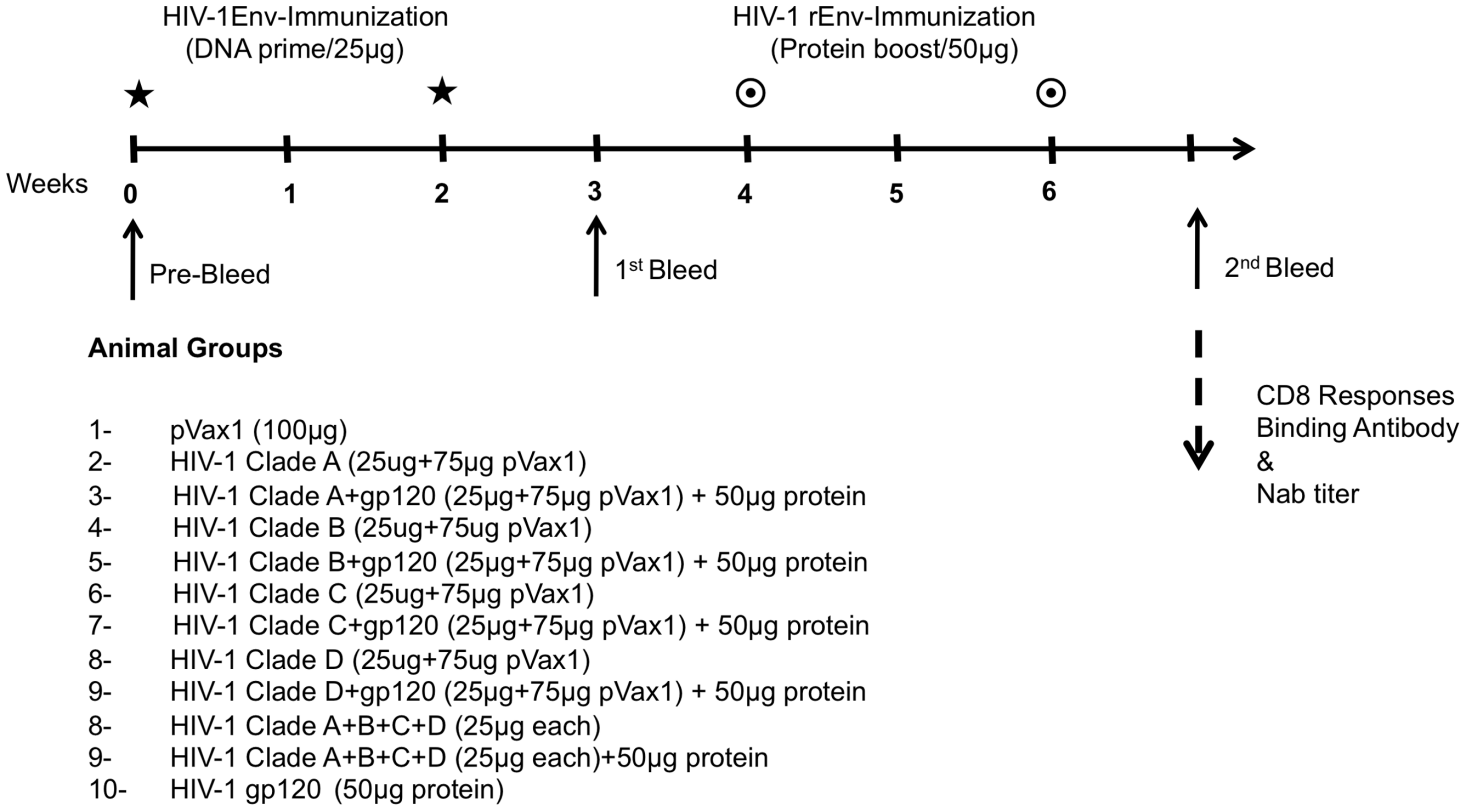
Design of animal groups for DNA prime-protein boost immunization study in BALB/c.

**Figure 3:**
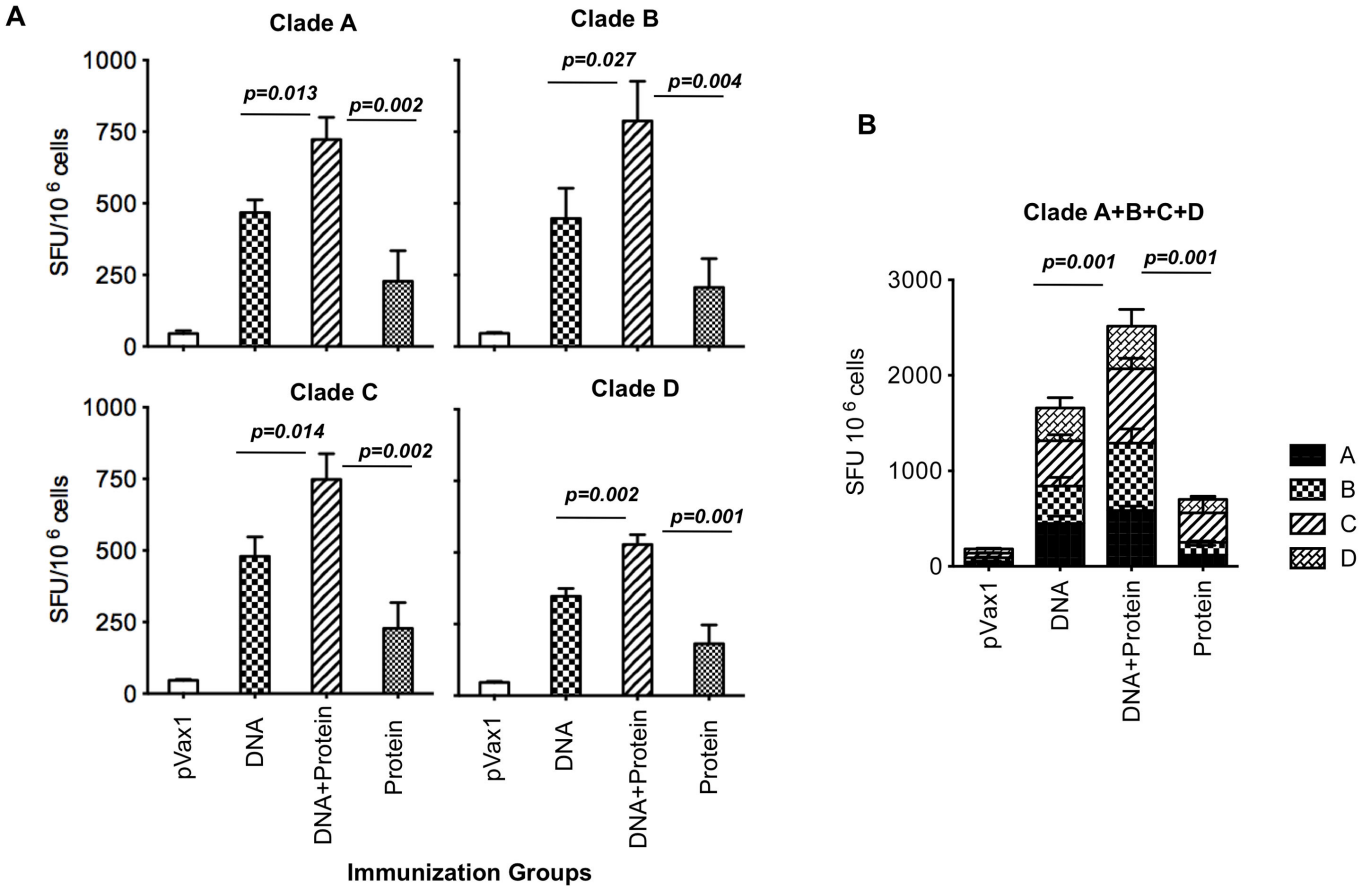
HIV-1 Env vaccines are potent inducers of cell-mediated immune response. The antigen-specific T-cell responses from a single plasmid (A) or combined plasmid (B) formulation were assessed by the IFN-γ ELIspot. Splenic T-cells were stimulated with BALB/c immunodominant Env peptide and IFN-γ spot forming cells were enumerated after overnight incubation. Results shown are the mean number of spot forming cells (SFC) ± SD for four animals/group with control SFC counts with background peptide subtracted.

Vaccine immunogenicity was assessed *in vivo* following immunization performed in mice. Mice were immunized with 25 μg each Env plasmid DNA using adaptive EP at week 0 and 2 (Figure 2). Mice receiving the protein boost were then immunized intramuscularly with 50 μg of HIV-1 MN gp120 formulated Titermix X-Gold adjuvant® at weeks four and six. One week post final immunization, mice were sacrificed and splenocytes were isolated to test the T-cell responses using an ELISpot assay, using the corresponding envelope peptide pools for stimulation. An increased number of Env-specific IFN-γ producing cells were observed in vaccinated animals, whereas control (pVax1) group splenocytes showed no response to Env peptide (Figure 3A). While DNA vaccination alone was superior to recombinant protein alone, the combination induced the best response in all cases. Interestingly, clade A, C and D group, DNA primes were boosted by a mismatched clade B antigen. Furthermore, mice immunized with the combined multi-clade DNA vaccine developed considerably higher IFN-γ production by CD8 T cells than mice injected with single-clade vaccines. There did not appear to be any antigen competition between envelopes (Figure 3B).

### Antigen-specific T-cells produce IFN-γ and IL-2 after DNA prime-protein boost immunization

**Figure 4:**
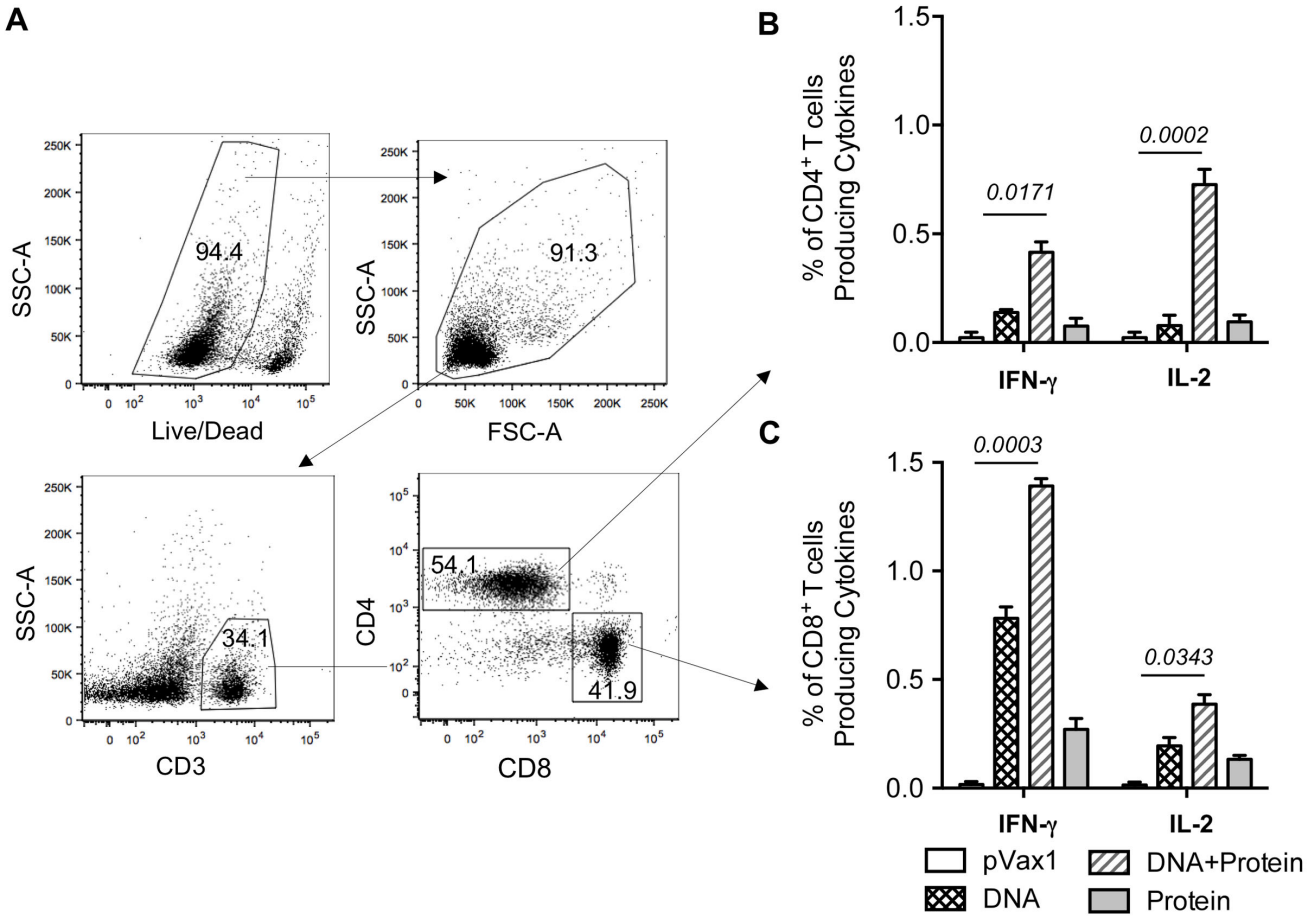
IFN-γ and IL-2 production in response to HIV-1 Env antigen. Intracellular cytokine staining with flow cytometry analysis of IFN-γ - and IL-2 expressing, Env-specific CD8^+^/CD4^+^ splenic T cells stimulated with Env peptides. Mice were immunized with multi-clade Env constructs and protein boost groups, and splenocytes were collected and cultured. (A) Representative flow cytometry data for splenocytes harvested from mice immunized with multi-clade DNA followed protein boost and stimulated for five hours with the envelope peptide. (B&C) Bar graph showing the number of Env-specific IFN-γ and IL-2 expressing (B) CD4^+^ and (C) CD8^+^T-cell responses generated by *in vitro* stimulation as described in Materials & Methods. Results are the mean ± SD for 4 mice per group (*n = 4*). Data presented in this figure are from one experiment representative of two performed.

Given the importance of polyfunctional T cell responses in controlling HIV-1 infection [8], [16], [42], we measured the ability of multi-clade specific T cell populations from immunized mice to secrete IFN-γ and IL-2 in response to envelope peptide pool stimulation. Functionally divergent Ag-specific T-cell populations are commonly defined by secretion of IL-2 by CD4 T cells populations and IFN-γ by CD8 T cell populations [4], [43], [44]. Therefore, we characterized Ag-specific CD8^+^ and CD4^+^ T-cell responses by simultaneous measurement of both IFN-γ and IL-2 secretion following peptide stimulation as described in Materials and Methods. We further analyzed the phenotype of the adaptive immune response elicited in multi-clade DNA+protein immunization groups by polychromatic flow cytometry, using FACS- based ICS, we evaluated IFN-γ and IL-2 after *in vitro* stimulation with HIV-1 Env peptide pools that covered the entire Env sequence. Our gating strategy for intracellular cytokine flow cytometric analysis is depicted in Figure 4A; this strategy allowed us to separate CD8^+^ or CD4^+^ T cells into subsets based on their ability to produce one or more cytokines. As shown in Figure 4B & C a multi-clade DNA prime and protein boost induced higher levels of IFN-γ/IL-2 secretion in CD8^+^/CD4^+^ T-cells. We observed that the percentage of Env-specific IFN-γ^+^ -secreting CD8^+^ T-cells was greater as that of IL-2^+^ secreting CD4^+^ T-cells, suggesting the induction of a more dominant CD8^+^ T-cell response observed in multi-clade vaccine. Another effect of the DNA prime-protein boost strategy was an increase in the CD8^+^/IFN-γ^+^ and CD4^+^/IL-2^+^ T-cell populations as compared to the CD8^+^/IL-2^+^ and CD4^+^/IFN-γ^+^ populations. Consistent with the results observed in the IFN-γ ELISpot, the combined DNA prime-protein boost generated more robust Env-specific T-cell responses. This result supports the conclusion that optimized DNA, in combination with EP delivery and vaccination with a protein boost, is able to induce expansion of functional Env-specific CD8^+^ and CD4^+^ T-cells.

### Effects of multi-clade formulations and protein boost regimens on Abs elicitation and B-cell activation

**Figure 5:**
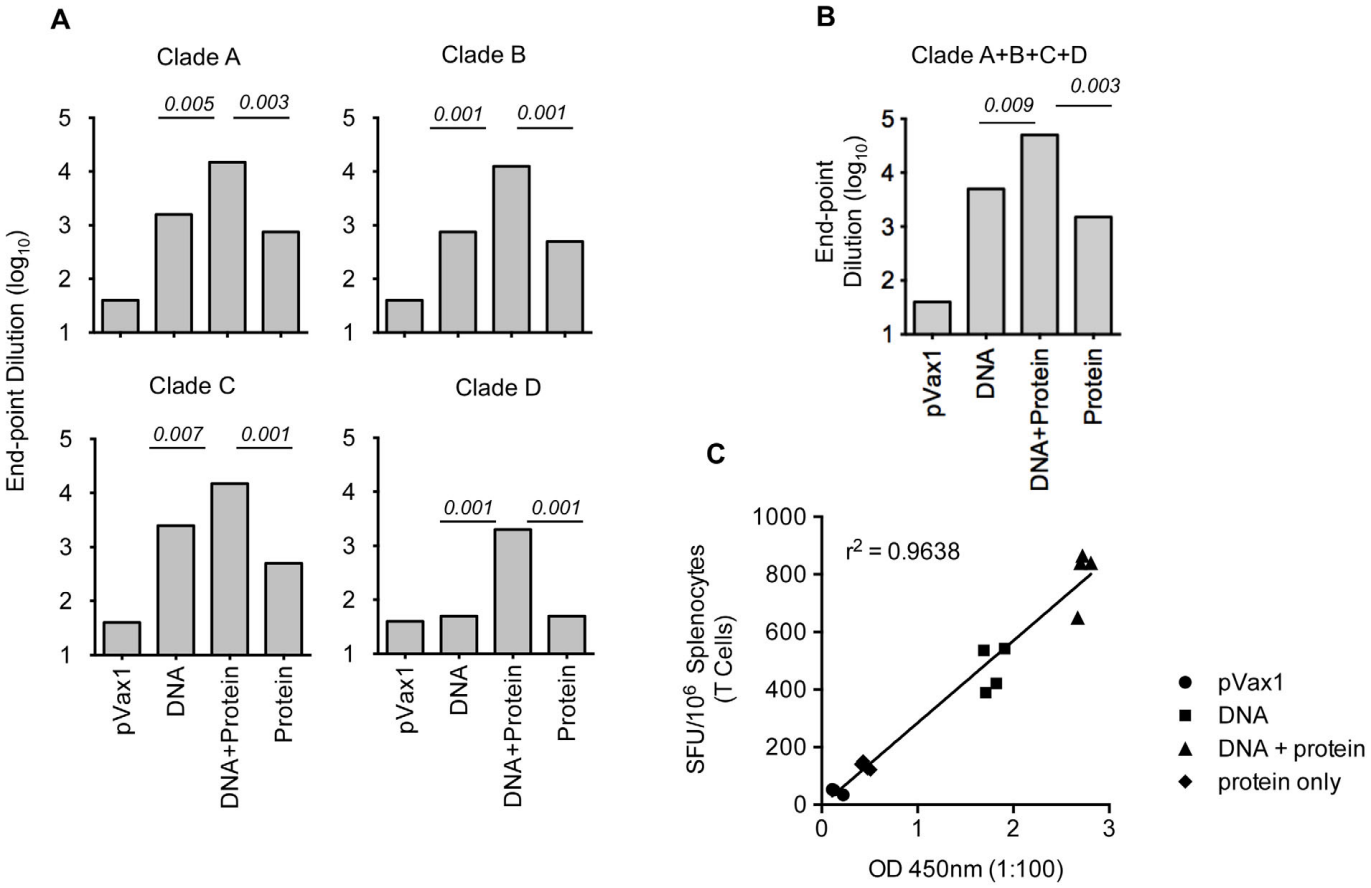
Characterization of antisera directed against HIV-1 Env. Binding of mouse antisera from DNA prime-protein boosts with subtypes A, B C and D envelope DNA and subtype B proteins. ELISA plates were coated with recombinant gp120 (subtype B) envelope glycoproteins. (A&B) End-point anti-gp120 IgG titers obtained from mice (*n = 4*) immunized with different Env immunogens as indicated, data shown titers at day 35, one week after the second protein boost. (C) Correlation between the binding antibody titers and SFU obtained by T-cell ELISpot assay.

Next, we wanted to test whether immunization with multiple Env variants induced higher titers of antibodies than immunization with any single construct alone. We measured the levels of Env-specific IgG production using pooled serum from the groups of mice by binding ELISA to HIV-1 Env. Surprisingly, DNA alone elicited higher antibody binding titers than single recombinant protein alone. However, a DNA prime-protein boost induced more robust binding titers than either DNA or protein alone (Figure 5A). More importantly, the multi-clade immunization elicited higher titers than did immunization with any single Env variant alone (Figure 5B). Titers from the multi-clade immunization were further enhanced with the addition of a protein boost. These studies illustrate that an improved humoral response is generated with a multi-clade DNA prime followed by recombinant protein boost. Further, we also observed a positive correlation between antibody titer to T-cell responses measured by ELISpot in the multi-clade DNA prime followed by a recombinant protein boost immunization strategies (Figure 5C).

**Figure 6:**
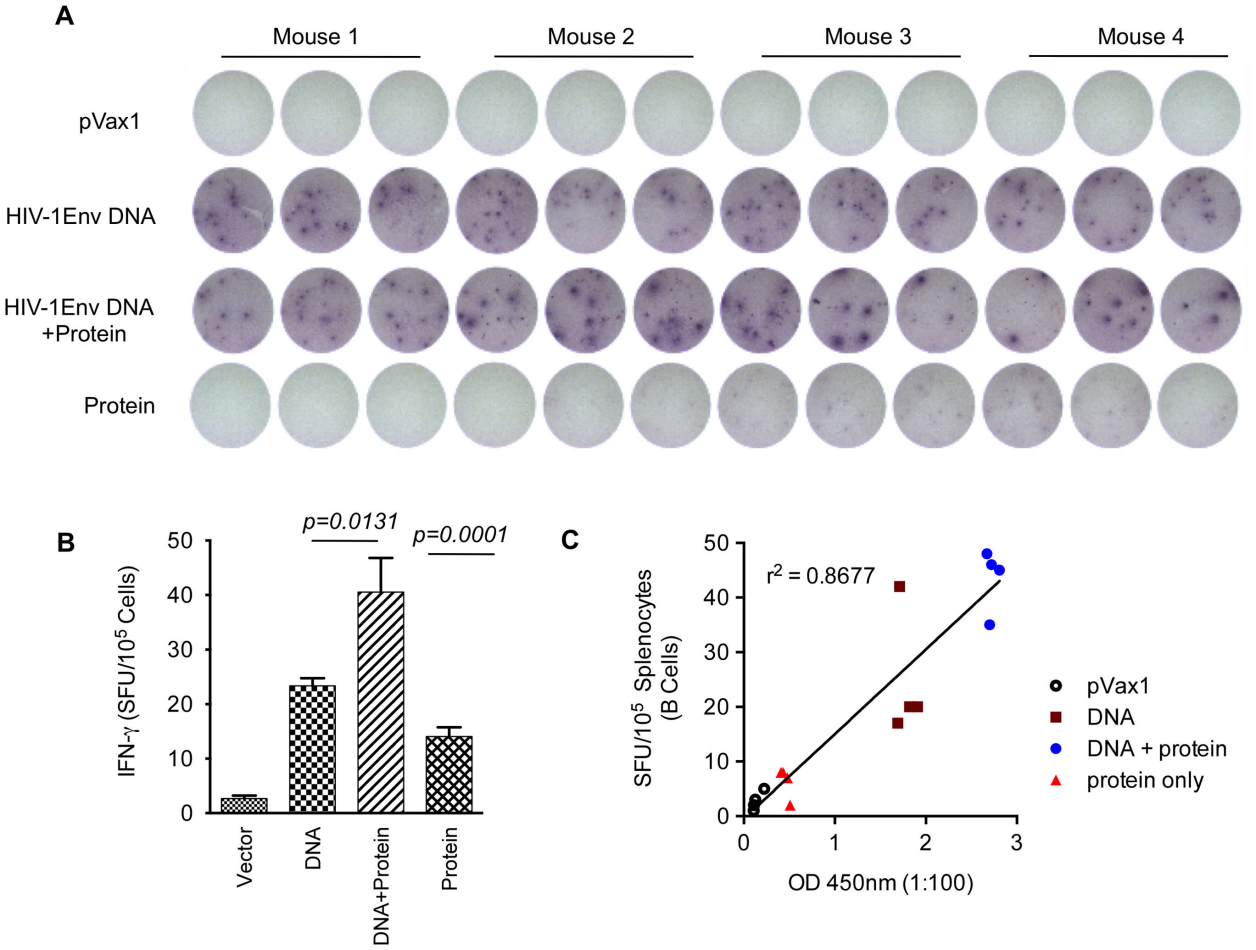
Detection of antibody secreting cells (ASCs). Groups of mice (*n = 4*) were immunized with the multi-clade constructs. (A) 96-well plates were coated with goat anti-mouse IgG in PBS and blocked overnight at 4°C. Approximate number of IgG producing B-cells was determined by ELISpot assay. (B) Representative plots of two individual experiments are shown; error bars represent standard deviation of at least three replicate wells. (C) Correlation between the binding antibody titers and SFU obtained by B-cell ELISpot assay.

To further understand the mechanisms underlying antibody production, B-cell ELISpots were performed to measure the frequency of vaccine-induced antibody secreting cells (ASCs). As indicated in Figure 6A&B, we observed higher levels of HIV-1-specific IgG producing B-cells following a DNA prime than protein boost. Also these results demonstrate that a DNA vaccine combined with EP can activate a robust humoral response as measured by IgG production by B-cell ELISpot. The B-cell response was enhanced by approximately 50% with the inclusion of a recombinant protein boost. Taken together, these data support the conclusion that optimized DNA alone can induce a strong humoral response and the effect is further enhanced by the addition of a protein boost. Further, we also observed a positive correlation between antibody titer to B-cell ELISpot in the multi-clade DNA prime followed by a recombinant protein boost immunization strategies (Figure 6C).

### Antibody levels in guinea pigs after DNA prime-protein boost immunization

**Figure 7:**
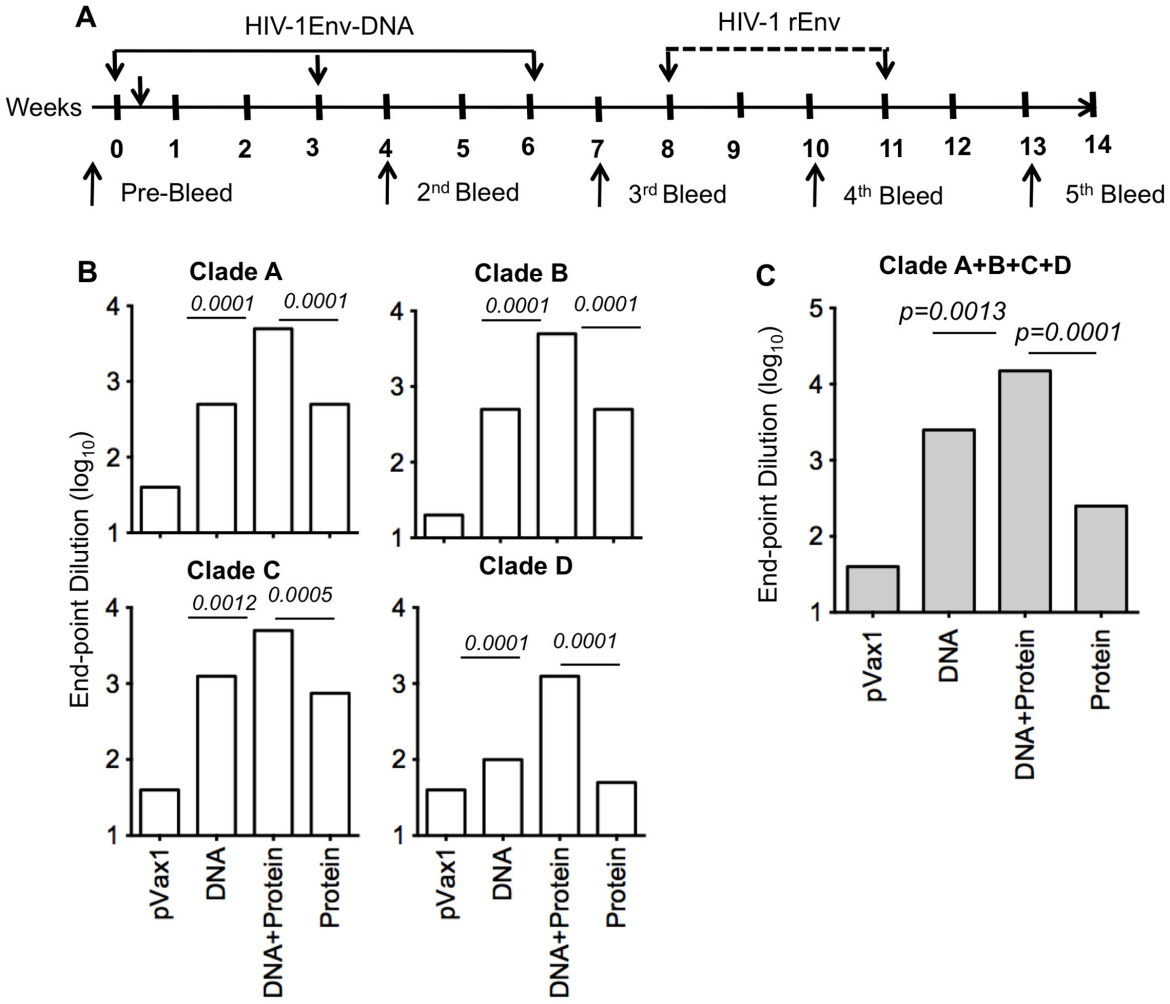
Serum IgG ELISA responses in guinea pig immunized with HIV-1 envelope. (A) Timeline for the DNA prime-protein boost immunization study in guinea pigs. Serum samples from the immunized and control guinea pigs were obtained as indicated. (B&C) Anti-gp120 antibody-binding titers were determined by ELISA two weeks after the first protein boost (*n = 5*). Data are presented as the mean endpoint titers ± D.

We further characterized the humoral response induced by the DNA prime-protein boost regimen in guinea pigs using individual envelope as well as multi-clade envelope with EP followed by protein boosts as indicated in Figure 7A.

Guinea pigs receiving the protein boost were then immunized intramuscularly with 50 μg of HIV-1 MN gp120 formulated Titermix X-Gold adjuvant® at weeks four and six. Guinea pigs allow for additional investigation of antibody responses since neutralization assays using TZM-Bl cells can be easily performed without the isolation of IgG as would be necessary in mouse serum. One week post final immunization, serum samples were measured for humoral immune responses. As seen in mice, the DNA prime-protein boost immunized guinea pigs exhibited the highest level of antibody responses as judged by binding ELISA. Similarly consistent with our mouse studies was the fact that multi-clade DNA alone resulted in superior levels of binding antibodies as compared to recombinant protein alone (Figure 7B&C).

**Figure 8:**
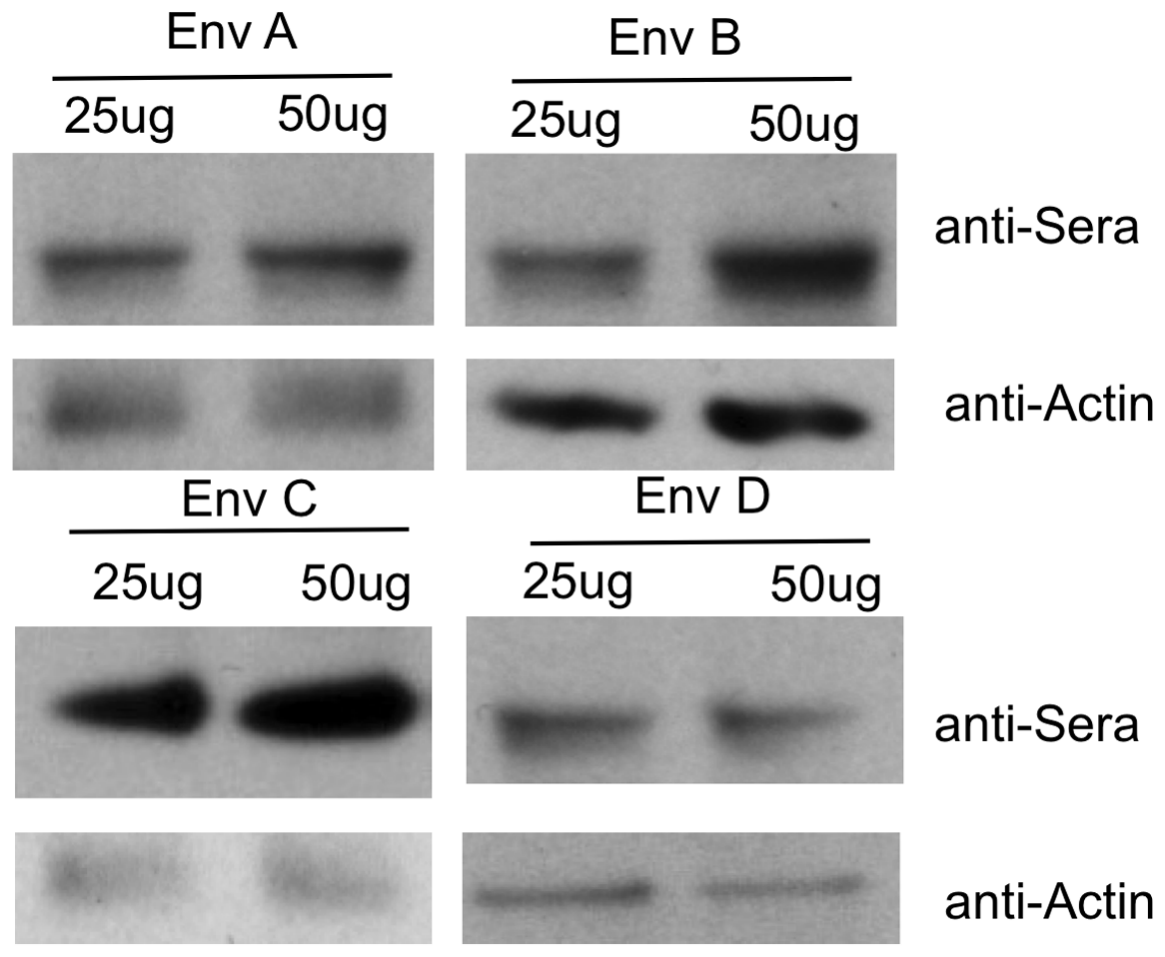
Characterization and specificity of anti-gp120 antibody raised in Guinea pig. Western blot analysis of anti-gp120 antibody response using sera from multi-clade prime and recombinant protein boost immunized guinea pigs. Cell lysates from 293T cells transiently transfected with HIV-1 Env plasmid as indicated and were loaded onto 10% SDS-PAGE and were analyzed by Western blot using sera from multi-clade Env immunized plus protein boost as the primary antibody at a dilution of 1∶500. Sera were collected two weeks after the final protein immunization.

To further characterize Env-specific responses induced in immunized guinea pigs, sera from multi-clade DNA prime and protein boost animals were tested for seroconversion using Western blot analysis to confirm specificity. Protein lysates were isolated from HIV-1 clade env transfected 293T cells and resolved using SDS-PAGE. Figure 8 shows that sera from multi clade Env-immunized plus protein boost bound to Env protein. Consistent with antibody analysis by ELISA, guinea pigs vaccinated with a multi-clade DNA vaccine plus protein boost exhibited a high level of cross reactivity with envelope proteins from different clades and demonstrate that the multi-clade DNA prime and protein boost vaccination procedure provided a technical platform to allow us to develop more effective HIV vaccines by using polyvalent Env formulations.

### Neutralizing antibody responses in guinea pigs

Sera from immunized guinea pigs were analyzed for the level and specificity of NAbs activity to define antibody functionality induced by a multi-clade DNA prime-protein boost. Guinea pigs sera were used because serum IgG does not need to be isolated as in mice for neutralization studies [45]. The serum neutralization titer was determined by assessing whether sera could neutralize 50% of the virus infection using the TZM-Bl cell assay system [37]. Sera were tested at in triplicate to test specificity.

**Figure 9:**
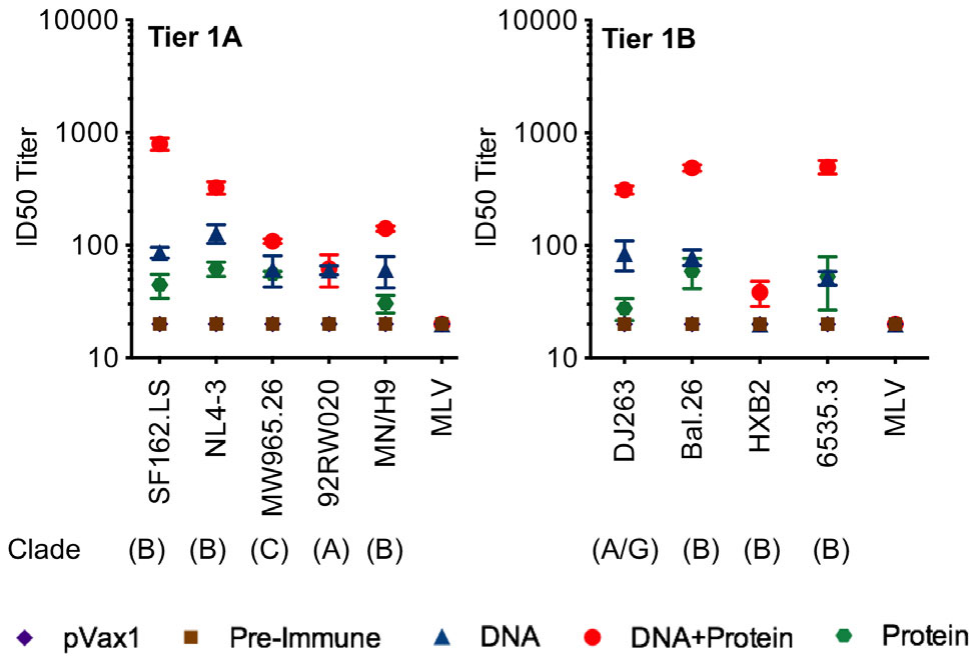
Neutralizing antibody titers against HIV-1 from immunized guinea pig sera. Guinea pig sera were collected two-Bl cells using a panel of envelope tier 1 pseudo viruses as described in Materials and Methods. Neutralization titers were defined by the sera dilution that achieves 50% inhibition of viral isolates (ID50).

We observed that immunization with a combination of Env clades A, B, C and D primed and protein boost was capable of generating NAbs using a standardized tier-1a and tier 1b panel of reference Env pseudoviruses for NAb assessment (Figure 9). Out of the nine viruses from different clades that were tested, six viruses were neutralized with pooled sera from the multi-clade DNA vaccination and protein boost group. In contrast, vaccination with recombinant protein alone exhibited very limited neutralization breadth: sera from this group showed detectable neutralization titers for only few viruses out of the nine viruses tested. Recombinant protein vaccination also showed limited cross-clade breadth.

Similar to the results from binding titer assays, the magnitude of NAbs production was enhanced by a DNA prime-protein boost vaccination protocol. The addition of a recombinant protein boost enhanced the titers generated by DNA alone by half a log for many of the viruses. These data suggest that a multi-clade DNA prime is capable of generating improved neutralization breadth with lower titer and the magnitude of this response is enhanced by the addition of a recombinant protein boost without decreasing breadth. Overall, the DNA prime-protein boost regimen yielded superior results in all antibody assays.

## Discussion

The major limitation of DNA vaccination in the past has been its relatively weak immunogenicity *in vivo*. Recent DNA studies using *in vivo* EP have suggested that this new generation of DNA vaccine is more immune potent and this result for cellular response has been observed in humans [7], [9], [15]. Prior studies by our lab and others exploring the use of first generation HIV DNA vaccines showed that weak binding antibodies to Env could be elicited through DNA technology mostly in small animals [24], [41], [46]. Antibody responses were enhanced by boosting with monomeric gp120 protein, but the NAbs were of low potency and exhibited little cross-reactivity [47]. We sought to improve upon these results by introducing changes to our vaccine platform that would redirect the immune system towards a stronger humoral response. These changes included enhanced consensus DNA vaccines with the potential to induce antigen expression on the cell surface, improvements in transfection efficiency and antigen presentation via adaptive electroporation, and the addition of a recombinant protein boost to further stimulate B-cell expansion.

The first DNA vaccines produced in the early 1990s were not able to induce reasonable levels of cell mediated immunity (CMI) in either non-human primates or humans [1], [4], [8], [16], [48], [49]. Through recent advances in DNA construct design and delivery methods, this approach now can produce strong CD8^+^ T-cell responses in these species [7], [10], [14], [19], [28], [50] and most importantly in two human clinical trails for HPV [9] and HIV strong T cell response [15] were induced in human by synthetic consensus optimized DNA delivered by adaptive EP. We followed up this encourage data in this prime boost studies presented here. We further characterized that vaccination with DNA plasmids encoding synthetic consensus envelope immunogens induce T-cell responses which are superior to protein alone. We also demonstrate that the most robust responses were achieved with a combined DNA prime-protein boost strategy, which particularly enhanced CD4 T-cell responses. When investigating the effects of a multi-clade DNA prime, we found that a combination of clades A, B, C and D improved T-cell responses as determined by both ELISpot and flow cytometry analyses (Figure 3 & 4). We considered the possibility that this phenomenon was the result of a dose effect since the total amount of DNA administrated to the multi-clade was 100 μg (25 μg of each clade) in comparison to 25 μg for the single-clade group. In order to control for this variable, we conducted a dosing study in which mice were given a range of doses for one single-clade vaccine. It was determined that doses >25 μg DNA did not significantly improve the immune response and therefore dosing alone could not explain the observed improvement (data not shown). A more likely explanation for this phenomenon is that a greater number of T-cells are activated from a multi-clade vaccine due to a greater number of unique epitopes presented by the vaccine. Interestingly, a multi-clade DNA prime-protein boost also appeared to drive a more functionally divergent T-cell response. Protein and DNA only vaccination produced roughly similar numbers of IFN-γ^+^/CD8^+^, and IFN-γ^+^/CD4^+^ T-cells, as well as similar numbers of IL-2^+^/CD4^+^ and IL-2^+^/CD8^+^ T-cells. In contrast, a multi-clade DNA prime-protein boost resulted in four times more IFN-γ^+^-secreting CD8^+^ T-cells than IFN-γ^+^-secreting CD4^+^ T-cells and higher IL-2^+^ -secreting CD4^+^ and CD8^+^ T-cells (Figure 4B&C). Further studies will be required to elucidate the exact mechanistic and phenotypic T-cell differences generated from the enhanced DNA prime-protein boost platform.

In addition to the induction of T-cell responses, consensus envelope vaccination also induced a robust humoral response. We observed that a multi-clade DNA vaccine induced higher antibody titers than the recombinant protein vaccine alone. Such results were surprising considering that recombinant protein has traditionally been the gold standard for antibody-based vaccines. These results may be due to the immunization with the recombinant protein used in this study. A different protein and adjuvant system may also have resulted in higher titers more consistent with those observed from our DNA vaccines. Despite the low antibody activity achieved with a single immunization of rgp120 alone, it served as an effective boosting agent, enhancing binding titers following a single- or multi-clade DNA prime. Between the single- and multi-clade DNA primes, greater antibody levels were achieved with the multi-clade formulation (Figure 7C). Similar to the T-cell responses, this might be a consequence of greater stimulation by a larger number of unique epitopes.

Another important feature of a future HIV vaccination is the breadth of the humoral response. Vaccination with clade A or clade C- DNA immunogens produced binding titers for clade B gp120 equal to those produced by a clade B DNA vaccine. We have similarly observed broad cross-reactive responses with some of our other synthetic consensus vaccines [29]. These broad responses are most likely attributable to the consensus design in which only the most common amino acid sequences are expressed, resulting in the presentation of conserved epitopes. The DNA platform itself may also contribute to the observed breadth, since the final gp140 protein product is more likely to be expressed in a functionally relevant trimeric form with natural glycosylation patterns. We are currently investigating the biological properties of consensus gp140 produced in host cells, focusing particularly on the oligomeric state and localization of the final protein product.

Similar to the data observed for antibody production in mice, the consensus DNA vaccine induced broader neutralizing titers when administered in guinea pigs. A consensus clade A, B, C and D vaccine induced titers against several primary clade viruses. In contrast, clade B recombinant protein induced only clade B neutralizing antibodies. Despite only inducing clade B NAbs when administered alone, recombinant protein delivered after a DNA prime was able to significantly improve the induction of clade A, B, C and D NAbs titers.

Our data support that enhanced DNA vaccines delivered by EP are useful for the induction of strong binding antibody responses against a broad range of viral strains. Combining DNA with a protein boost elicited enhanced NAbs activity against a panel of viral isolates. Thus, the data provide important support that enhanced adaptive EP delivered DNA prime-protein boost is an important strategy for delivering polyvalent Env-based HIV vaccines aimed at improving breath. Further immunization exploration of the immune response induced by diverse HIV DNA cassettes in combination with multiple protein boosts will likely provide important information of relevance to HIV vaccine development.
